# Supplementary material for: Are carbon emissions trading and green financial instruments synergistic? -Comprehensive quantitative research based on content analysis
Source: PLoS One. 2024 Mar 7;19(3):e0298601. doi: 10.1371/journal.pone.0298601 (PMC10919601; doi:10.1371/journal.pone.0298601)
Supplement: S1 Data — (ZIP) [file pone.0298601.s001.zip › Data/地级市Z_F工作报告.zip!/║╙─╧/░▓╤⌠╩╨/└┤╫╘╣1⁄2╓┌║┼í╛┬φ┐╦╩2╛▌═°í┐_2010.docx]

各位代表：

    现在，我代表市人民政府，向大会报告政府工作，请予审议，并请各位政协委员提出意见。

    一、2009年工作回顾

    2009年是新世纪以来我市经济社会发展最为困难的一年。受国际金融危机严重冲击，年初我市经济增速大幅下滑，企业经营陷入困境，农民工大量返乡，财政收支矛盾突出，加之严重自然灾害的发生以及长期积累的体制性、结构性矛盾集中显现，经济社会发展面临前所未有的压力和挑战。面对异常困难的局面，在省委、省政府和市委的坚强领导下，全市上下深入贯彻落实科学发展观，同心协力，迎难而上，奋发进取，扎实工作，圆满完成了市十二届人大一次会议确定的各项目标任务，在逆境中实现了经济社会平稳较快发展。

    ——综合实力显著提升。初步测算，全市生产总值完成1106亿元，增长12.2%，人均生产总值突破3000美元。地方财政一般预算收入55.2亿元、支出121.7亿元，分别增长10.3%和27.9%。

    ——工业经济企稳回升。全市规模以上工业增加值532亿元，增长15.2%；实现利润146.9亿元，增长10.3%。尤其地方工业增速位居全省前列。

    ——粮食生产再创新高。粮食总产在大灾之年达到333.1万吨，连续7年丰产丰收。滑县实现全国粮食生产先进县标兵七连冠。

    ——投资消费同步增长。全社会固定资产投资完成741亿元，增长30.9%；城镇固定资产投资完成622.6亿元，增长31.5%。社会消费品零售总额完成292.5亿元，增长19%。

    ——人民生活持续改善。全市城镇居民人均可支配收入14809元，增长8.6%；农民人均纯收入5595元，增长7.8%，均实现预期目标。

    一年来，我们突出抓了以下工作：

    （一）战危机，促增长，实现经济平稳较快发展。面对复杂严峻的经济形势，我们科学应对，及时把握不同时段经济运行的突出矛盾和问题，紧紧抓住市场开拓、企业服务、投资拉动等关键环节，出台了一系列针对性强的政策措施，形成了应对危机的“组合拳”，有效遏制了经济下滑趋势，全年经济运行成功实现“V”形反转。积极服务企业。围绕提振信心、破解难题、优化环境，深入开展“企业服务年”活动。大力开展企企对接，帮助企业开拓市场，先后出台了20多项政策措施，一批制约企业发展的问题得到有效解决。着力促进投资。深入实施项目带动战略，建立完善了重大项目联审联批和“绿色通道”制度。争取中央和省投资项目549个，总投资39.4亿元。强力推进“8155”投资促进计划，153个省、市重点项目完成投资126亿元。安彩高科560万平方米光伏玻璃、顺城90万吨捣固焦等21个带动性强的大项目建成投产。不断扩大消费。培育消费热点，优化消费环境，大力实施家电下乡、汽车摩托车下乡、汽车以旧换新和住房消费等政策。全年销售家电下乡产品21.4万台（部），兑付补贴资金4986万元。商品房销售面积244.1万平方米，增长27.9%。文化、旅游、娱乐、电子信息等消费保持较快增长。保障要素供应。推动银企合作，金融支持经济发展力度明显增强。全市金融机构新增贷款114亿元，较上年增长一倍。新设立29家中小企业担保机构，融资担保10.2亿元。加强煤电油气运调节，较好地保障了重点项目建设和正常生产生活需要。

    （二）调结构，促转型，增强经济社会发展后劲。在战危机、保增长的同时，更加注重经济发展方式转变，跨越发展的基础日益坚实。大力推进产业集聚区建设。聘请德国罗兰？贝格公司编制了《安阳市城市总体功能定位、主导产业确定及空间布局优化》规划，研究制定了《加快安阳市产业集聚区科学发展若干政策意见》等政策措施，9个省级产业集聚区总体发展规划在全省率先通过评审，累计完成投资155亿元，载体功能逐步显现。积极构建现代产业体系。重点培育壮大6+1支柱产业，工业固定资产投资385.6亿元，增长50.9%，占全行业投资比重61.9%，较上年提高8个百分点。继续推进“工业振兴工程”，全面实施产业振兴计划，改造提升传统产业，培育壮大新兴产业，全力打造新能源谷。成功举办“中国光伏产业（安阳）高层论坛”，荣获“中国光伏产业示范基地”称号。中升多晶硅、新能光伏、欧美亚光伏、凤凰光伏等一批新能源企业快速成长。金融、物流、信息、房地产等现代服务业蓬勃发展，华强建国酒店、彰德府商业文化步行街等一批三产项目顺利推进。努力构建自主创新体系。全面实施《安阳市自主创新体系建设和发展规划》。安钢帘线钢达到国内顶尖水平，高强度汽车用钢卷板填补了省内空白。13家企业技术中心被确定为省级技术中心，4家企业被确定为省创新型企业，8项科技成果获得省科技进步奖。全市高新技术产业增加值达到32.2亿元，增长29.7%。荣获全国科技进步先进市、国家知识产权试点市称号。加快发展县域经济。县域生产总值826亿元，增长15.3%，占全市生产总值比重达74.7%；财政一般预算收入22.7亿元，增长15%，占全市比重达41.2%，安阳县、林州市财政收入均突破8亿元。汤阴县成功创建“国家新型工业化产业示范基地”，进入“中国农业产业经济发展百强县”行列。滑县产业集聚区、内黄县中原瓷都建设积极推进。全市12个乡（镇）入围河南省百强乡镇，其中水冶镇连续5年综合实力排名第一。扎实推进节能减排。全市万元生产总值能耗下降6.02%，化学需氧量和二氧化硫排放量分别控制在4.6万吨和10.1万吨以下，空气质量优良天数达到324天，省控地表水断面达标率首次达到90%以上。

    （三）打基础，促增收，推动农村经济全面发展。认真落实强农惠农政策，大力实施“四大兴农计划、三大惠民工程、十大农业产业链开发、十大农业增效技术和林业生态市建设”，农业农村发展形势良好。大力推进农业产业化。全市各类农业产业化企业达到435家，其中年销售收入1000万元以上148家，超亿元29家，产值60.8亿元，安置从业人员4.9万人，培植农业专业合作社1100家。积极发展高效农业。新增亩均效益3500元高效农业面积15.3万亩，新建扩建高效农业示范园区46个，内黄县被国家命名为蔬菜生产基地区。新建畜禽养殖密集区118个，大中型养殖企业77个。切实加强新农村建设。“十大标兵村”和130个试点村建设成效显著，乡村清洁工程和农村社区建设试点工作进展顺利。新建大中型沼气池96座，新增沼气用户4.8万户。解决了26.1万人的安全饮水问题。完成农村劳动力转移培训4万人。新建、改造农村公路1040公里。完成86个贫困村整村推进任务，解决了4.8万农村贫困人口脱贫和温饱问题。新增节水灌溉面积24.4万亩，南水北调安阳段和小南海、弓上、双泉等水库除险加固工程积极推进，连续16年夺得“红旗渠精神杯”。加快林业生态市建设，新增营造林面积48.8万亩。

    （四）谋长远，促协调，积极构建现代城镇体系。坚持中心城市带动，加强城市规划建设和管理，城镇化水平进一步提高。着力做好现代城镇体系规划。新一轮城市总体规划、土地利用总体规划修编完成，并报国家审批。积极推进188平方公里安阳新区规划建设，“六路三片区”等19项专项规划编制纳入市区整体规划。全市城乡一体化发展战略规划及各县（市、区）城镇总体规划全部完成。全市建制镇、乡集镇总体规划和近期建设规划修编完成率达85%，村庄规划完成率达75%以上。中心城市、中小城市和小城镇各具特色、竞相发展的新格局初步形成。强力实施“城建三年计划”。总投资530亿元的“4298”工程，第一批、第二批74个项目已开工47个。“七路一网”等16个项目已完工，累计完成投资40.6亿元。城市水系规划建设全面铺开，引岳（岳城水库）入安生态调水工程正式通水。创建“国家园林城市”圆满成功。数字化城管顺利启动。建设国家级交通枢纽城市取得积极进展，林长高速、鹤辉高速林州段全面开工，安林高速西站出口、大广高速慈周寨站出口连接线工程建成通车，京港澳高速安阳段扩建工程扎实推进，石武高铁安阳段建设进展有序，山西中南部铁路通道开工建设。安阳至北京动车组开通运营。安阳火车站整修到位。航空港、陆路港建设取得实质性进展，与邯郸市、日照市分别签署了合作协议。切实加快县城和小城镇建设。大力开展优胜杯竞赛活动，县城和小城镇承载功能得到提升。全市城镇化率达38.9%，较上年提高1.6个百分点。

    （五）创品牌，促提升，加快发展文化旅游产业。坚持 “三阳开泰”旅游发展战略，文化旅游产业逆势上扬，成为拉动我市经济社会发展的新引擎。全市文化产业增加值增速超过生产总值增速，累计接待国内外游客1430万人次，旅游总收入47.9亿元。文化体制改革进展顺利。积极推进文化体制改革试点市工作，8家文化院团完成改制任务，市文化市场综合执法支队挂牌成立。文化旅游活动亮点纷呈。成功举办滑翔伞世界杯热身赛、颛顼帝喾陵祭祖节、殷商文化旅游节、世界文字论坛、世界客家人播迁路圣土采集和归集仪式等大型节庆活动。文化旅游项目影响深远。世界上唯一以文字为主题的博物馆——中国文字博物馆震撼开馆。曹操高陵成功发掘，轰动海内外，成为2009年全国6大考古发现之一。殷墟国家大遗址公园建设积极推进，三杨庄汉代遗址博物馆建成并对外开放。马氏庄园直接创建为4A级景区，成为旅游新热点。太行大峡谷被评为国家地质公园。文化艺术创作精品迭出。电影《别样警花》央视热播，电视剧《家在洹上》、《今生欠你一个拥抱》受到好评，豫剧《野牯岭》演出成功，专题片《中国豫剧》开机拍摄，河南坠子《观花灯》、戏曲小品《南邻北院》省内获奖。

    （六）抓改革，促开放，激活经济发展内生动力。坚持把改革开放作为应对危机、实现跨越发展的根本途径，着力增强经济社会发展的动力和活力。各项改革深入推进。77家国有企业启动了改革程序，6家基本完成改制。河南煤化集团控股安化集团，益康制药和四川科伦集团实现战略重组。医药卫生体制改革正式启动。市、县政府机构改革进展顺利。交通银行落户安阳，民生证券安阳营业部开业。财税体制、行政审批制度、投资体制等各项改革也都迈出新步伐。对外开放水平不断提高。积极开展招商引资暨旅游推介“百日会战”活动，成功举办第七届全国特种养殖种植技术成果博览会、美国硅谷安阳光伏推介洽谈会。积极组织参加中原文化港澳行暨豫港澳经贸洽谈会、第四届中博会等一批大型经贸活动。加强与央企等国内外优势企业合作，促成了一批战略合作重大项目。全市实际利用外资1.2亿美元，利用域外资金272.8亿元，分别增长15.6%和19%。

    （七）保民生，促稳定，营造良好社会发展环境。去年全市财政投入十个方面93件实事的资金达32.4亿元，增长45.9%，高于一般预算支出增幅18个百分点。一笔笔惠民资金，一项项利民政策，使广大人民群众得到了众多实惠。就业再就业工作成绩显著。大力实施全民创业，突出抓好高校毕业生、下岗失业人员和农民工就业。全市城镇新增就业10万人，再就业3.7万人，其中困难群体再就业1.3万人。农村劳动力转移就业158.6万人。零就业家庭保持动态为零。社会保障工作进一步加强。建立了被征地农民社会保障制度，启动了新型农村养老保险试点和基本医疗保险市级统筹试点。城镇居民医保参保范围进一步扩大，新农合参合率达98.1%。企业退休人员基本养老金、城市低保和优抚对象补助水平进一步提高，农村低保扩面提标工作进展顺利。各项社会事业全面发展。继续免除义务教育阶段学生学杂费，改造农村校舍12所。安阳职业技术学院组建，并面向社会招生。安阳师院人文学院新校区开工建设，河南卫校、安阳幼师、市中医药学校搬迁工作有序推进。公共卫生服务体系逐步健全，社区卫生服务功能日益完善，甲型H1N1流感、手足口病等重大传染病防控工作取得显著成效。体育训练中心建设进展顺利，全民健身活动蓬勃开展。人事人才工作得到加强。人口和计划生育工作成效显著。

精神文明和民主法制建设进一步加强。扎实推进市民素质提升工程，积极开展各类道德实践活动和群众性精神文明创建活动，公众道德水平和城乡文明程度有所提升。成功创建第二届全省文明城市。扎实开展深入学习实践科学发展观活动，持续推进政府机关“两转两提”，各级政府机关服务效能和工作水平进一步提高。坚持依法治市，严格依法行政，自觉接受人大法律监督、工作监督和政协民主监督，认真办理人大代表议案、建议和政协委员提案。深入开展“安全生产年”活动，全市各类事故起数和死亡人数分别下降13.7%和16.1%。食品药品安全保障水平继续提高。加强社会治安防控体系建设，依法做好信访工作，社会大局保持稳定。市信访接待服务中心建成并投入使用。市档案馆顺利晋升国家一级档案馆。双拥共建工作深入开展，军政军民团结进一步加强。国防教育和国防后备力量建设取得新成效，人防工作得到加强。切实纠正部门和行业不正之风，反腐倡廉工作取得新进展。不断加强老区建设。依法保护妇女儿童合法权益。老龄和关心下一代工作健康发展。统计、审计、史志、气象、地震、经济研究、外事侨务、民族宗教等工作取得新成绩。对口支援四川江油灾后重建工作进展顺利。

各位代表！

    过去一年，在挑战和困难面前，我们以特殊之举破解困难之局，不仅经受住了严峻考验，掌握了经济工作的主动权，同时也为今后工作特别是驾驭复杂局面、促进经济平稳较快发展积累了宝贵经验。一是始终坚持以科学发展观统领经济社会发展全局。面对严峻挑战，我们紧紧抓住发展第一要务不动摇，以人为本，统筹兼顾，更加注重经济转型，不仅保持了经济平稳较快增长，而且为我市长远发展奠定了基础。二是始终坚持在应对挑战中抢抓发展机遇。我们辩证地看待形势，既做到迎难而上、沉着应对，又看到危中有机，积极争取国家投资，顺势推进企业重组、节能减排等工作。正是由于我们直面困难，勇对挑战，在逆境中捕捉和把握发展机遇，才取得了应对危机的重大胜利。三是始终坚持用改革开放破解发展难题。我们充分利用市场需求约束加剧形成的倒逼机制和要素约束减弱的有利时机，着力深化文化、医药卫生等重点领域和关键环节改革，建立有利于科学发展的体制机制；充分发挥文化旅游资源优势，强化宣传，扩大开放，安阳知名度和影响力进一步提高。改革开放在危机中没有停步而是加快推进，经济社会发展动力和活力进一步增强。四是始终坚持把改善民生作为各项工作的出发点和落脚点。在保增长的同时，我们更加注重保障和改善民生，着力解决就业、就学、就医、住房和社会保障等关系群众切身利益的问题，群众生活在总体上不仅没有受到严重影响反而得到进一步改善。五是始终坚持协调联动打总体战。充分凝聚各方面智慧和力量，调动各方面积极性和创造性，既大胆负责、勇于担当，又加强合作、协同作战，全市上下形成了迎难而上、拼搏进取的强大合力。实践证明，无论遇到任何艰难险阻，只要我们坚定不移地走科学发展之路，坚持不懈地团结奋斗，就一定能化压力为动力、变被动为主动，不断夺取经济社会发展新胜利。

各位代表！

    在罕见的困难和挑战面前，我市经济社会发展取得这样的成绩极其不易。这是市委正确领导的结果，是市人大、市政协大力支持的结果，也是全市人民和衷共济、团结奋斗的结果。在此，我代表市政府，向奋斗在全市各条战线上的广大工人、农民、干部、知识分子、解放军指战员、武警官兵、政法干警以及各界人士，致以崇高的敬意！向所有关心、支持我市现代化建设的港澳台同胞、海外侨胞、国际友人，表示衷心的感谢！

    在肯定成绩的同时，我们也清醒地看到，我市经济社会发展还存在一些不容忽视的矛盾和问题。一是发展方式粗放、经济结构不合理、竞争力不强等问题在危机中愈加凸显，部分行业和企业生产经营仍较困难，外贸出口预期目标没有完成。二是社会事业历史欠账较多，教育、医疗、就业等一些事关人民群众切身利益的问题还没有得到很好解决。三是县区之间发展不平衡，市级财源建设不足，国企改革和个别经济、城建项目推进力度不够，与全市人民的期待还有一定差距。四是个别部门和单位，办事拖拉，效率不高，为企业、为基层服务的意识不强，发展环境有待进一步优化。对此，我们一定高度重视，在今后的工作中认真加以解决。

    二、2010年总体要求和奋斗目标

    2010年是实施“十一五”规划的最后一年。做好今年的工作，对夺取应对危机全面胜利、促进经济平稳较快发展、为“十二五”规划启动实施打好基础，具有十分重要的意义。

    今年政府工作的总体要求：全面贯彻党的十七大和十七届三中、四中全会精神，以邓小平理论和“三个代表”重要思想为指导，深入贯彻落实科学发展观，扭住工业，抓好农业，加快发展第三产业，大力实施项目带动战略，加强产业支撑、城市建设支撑、交通运输支撑和城市软实力支撑，推动经济发展方式转变和经济结构调整，推进改革开放和自主创新，着力改善民生和维护社会和谐稳定，保持全市经济社会跨越式发展的良好态势，努力在建设豫北区域性中心强市上取得新突破。

今年经济社会发展的主要目标：生产总值增长10%；财政一般预算收入增长10%；规模以上工业增加值增长13%；全社会固定资产投资增长22%；社会消费品零售总额增长17%；居民消费价格涨幅控制在3%左右；外贸出口增长15%；实际利用外资增长15%以上；城镇居民人均可支配收入增长8%；农民人均纯收入增长7%；单位生产总值能耗降低5.33%；人口自然增长率控制在6.45‰以下；新增城镇就业6.5万人以上，城镇登记失业率控制在4.5%以内。

    在实现上述目标的同时，市委、市政府今年还要全力实施十项民生工程，为全市人民办好十个方面实事，请一并审议。

    围绕上述目标和要求，必须牢牢把握以下六项原则：一是既要保持发展速度，又要调整产业结构、转变发展方式，实现发展的可持续性。跨越式发展包括量的扩张和质的提升，必须在继续保持经济平稳较快增长的同时，加快经济结构调整和发展方式转变，实现速度、结构、质量、效益的有机统一，为经济长期持续较快发展提供支撑。二是既要重视投资拉动，又要扩大消费、增加出口，实现发展的均衡性。继续抓住有效需求不足这一主要矛盾，在优化结构的前提下持续扩大投资规模，力求通过重点项目建设促进经济增长，带动产业升级、民生改善和社会事业发展，逐步实现投资、消费、出口协调拉动。三是既要优化和提升传统产业，又要引进和培育新兴产业，实现发展的科学性。把自主创新作为发展战略性新兴产业的有效途径，抓住世界科技进步和产业结构调整的机遇，改造提升传统产业，加快培育战略先导产业，抢占未来竞争高地，全面提升产业竞争力。四是既要抓好工业振兴，又要加快农业产业化和城镇化步伐，实现发展的统筹性。在强化农业基础地位、稳定粮食生产的同时，坚持走工业化和城镇化的道路，推动“三化”相互促进、协调发展。五是既要深化改革，又要扩大开放，实现发展的创新性。改革开放的成效决定着跨越式发展的成败，必须抓住有利时机，强力推进重点领域和关键环节改革，坚定不移地实施开放带动主战略，努力创造充满活力、富有效率、更加开放的体制机制环境。六是既要保持经济增长，又要关注和改善民生，实现发展的协调性。把保障和改善民生作为发展经济的最终目的，着力民生、着力民心，进一步加大投资力度，解决好群众的生产生活问题，切实做到发展为了人民、发展依靠人民、发展成果由人民共享。

    三、2010年工作安排

    完成今年各项目标任务，必须按照“重在持续、重在提升、重在统筹、重在为民”的要求，把坚持“三保”、突出转型、强化态势作为重点，把落实完善、创新机制、提高能力作为保障，积极推进“一个载体、三个体系”建设，加快实施“三大工程”和“十大行动计划”。

    （一）扎实推进经济平稳较快发展。进一步增强经济运行调控调节的预见性、针对性和科学性，积极扩大内需，优化需求结构，巩固经济持续回升向好态势。深入开展服务企业活动。健全政策导向、市场开拓、融资服务、困难企业帮扶等10项长效机制，推动企业服务常态化。建立健全企业服务中心，完善服务措施，提高服务效率和水平。加强重点项目建设。继续深入实施项目带动战略，扎实推进“重点项目双百工程行动计划”，在工业、能源交通、社会事业、商贸物流等领域，建设100个产业升级和基础设施项目，全年完成投资100亿元以上。突出抓好安钢铁前配套、凤宝年产50万吨石油井管、大众煤矿年产120万吨扩建、500千伏安阳西二期输变电等重点项目建设。加快发展现代服务业。充分发挥服务业发展引导资金作用，突出抓好现代物流、金融、房地产等服务业发展，积极推进义乌小商品城、众品生鲜物流加工配送中心、开发区铁路专用线和物流园区等项目建设，加快发展住宿餐饮、社区商业、物业、家政、休闲、文化等服务性消费。积极扩大消费需求。全面落实国家调整收入分配政策，健全企业最低工资制度、农民工工资支付保障机制，提高企业退休人员基本养老金、部分优抚对象待遇和城乡居民最低生活保障水平。落实好义务教育学校绩效工资政策。继续实施万村千乡、双百市场和农超对接等活动，加快农贸市场改造，进一步做好家电下乡、汽车摩托车下乡等工作。做好经济运行调控调节。加强对重点行业、骨干企业和主要产品的监测分析，组织好煤电油气运等供需衔接，保障企业要素供给。进一步提高财政调控能力，优化财政收支结构，加强财政资金统筹安排与整合，加大对“三农”、民生等重点领域支持保障力度。推动政府投融资平台健康有序发展，落实各项税费优惠政策，进一步减轻企业负担。依法规范税收征管和非税收入管理，严厉打击偷骗税行为。加强市场监管，努力保持物价合理水平。

    （二）扎实推进发展方式转变。加快产业结构调整，提升自主创新能力，努力在发展中促转型，在转型中谋发展。大力实施“产业集聚区建设工程”。加快市高新技术开发区晋升国家级开发区进程。规划建设安东新城示范引领区和安西循环经济试验区，推动“三化”互动、融合发展。科学编制园区总体规划，全面落实支持省级产业集聚区和专业工业园区发展的优惠政策，加快规划建设市属工业园区，真正形成一批基础设施完善、产业特色突出、公共服务健全的产业集聚区和工业园区。力争全年新开工项目128个，投产项目76个，完成投资208亿元，实现营业收入超过700亿元，促进集聚发展态势初步形成，推动整体竞争能力明显提升。着力壮大战略支撑产业。继续深入实施“工业振兴工程”，进一步改造提升传统产业，积极培育战略新兴产业。强力推进安钢冷轧、安化乙二醇、利源焦化200万吨捣固焦、三丰铜业10万吨超薄铜板带等七个投资10亿元以上重大项目和重机集团采煤机、轻机造纸设备、锻压机械数控设备、嘉和机械脱硫环保设备等装备制造、节能环保、高科技项目建设，提升优化我市产业层次。加快开发区装备制造基地、内黄县中原瓷都、汤阴县医药工业园、滑县服装工业园建设。完善新能源产业规划，支持安彩太阳能电池、嘉能能源二甲醚等新能源项目建设，着力打造新能源谷。积极优化企业结构。支持优势企业加快兼并重组步伐，依托安钢、沙钢永兴等企业组建大型钢铁集团，打造高水平精品钢基地。以顺成、鑫磊为龙头，组建税收超亿元的大型煤焦集团，打造千万吨级煤化工和煤电一体化基地。大力发展中小企业，落实各项扶持政策，加快建立多层次中小企业信用担保体系，努力解决融资难问题。着力提升自主创新能力。积极实施“自主创新行动计划”，突出自主创新主体，建设自主创新平台，不断增强自主创新能力和科技支撑经济社会发展的能力。新建20个市级以上研发中心，其中省级以上9个。新建5个市级以上重点实验室，支持中棉所、安阳师院、安阳工学院等单位申报建设国家、省重点实验室。大力实施质量兴市战略，引导激励企业积极争创省长质量奖、市长质量奖和名牌优质产品，努力将核心技术和知识产权专利化、标准化。加强节能减排和生态环境建设。编制实施节能环保产业规划，完善节能减排体制机制。严格执行“两高”产业淘汰标准，推广先进节能技术和节能设备，在电力、煤炭、有色金属等8大重点高耗能行业实施节能攻坚。积极发展循环经济和低碳经济。强化环境保护和生态建设。切实加强饮用水水源保护，建立健全水环境预警制度，从今年起建立水环境生态补偿机制。

    （三）扎实推进“三农”工作。强化支农惠农政策，认真实施“兴农行动计划”，加快发展现代农业，推进新农村建设，促进农业增效、农民增收。巩固提高粮食生产能力。突出抓好东部粮食核心区建设，全年粮食种植面积稳定在800万亩以上，力争总产实现新突破。全面启动农业发展改革引领试验区建设。突出资源优势和发展特色，打造亮点、探索机制，积极规划启动6个农业发展改革引领试验区建设。深入推进农业结构调整。加快实施百万亩高效农业开发计划，抓好30个百亩园、千亩方、万亩区高效农业示范园区建设，新增亩均效益3500元以上的高效农业面积15万亩。加快现代畜牧业发展。新增畜禽养殖密集区50个，新建大中型规模养殖加工企业50家，使大中型规模养殖加工企业和畜禽养殖密集区达到1000家。积极推进农业产业化经营。新建扩建龙头企业25家，继续抓好农业产业化示范县（市、区）、乡（镇）和特色农村经济先进乡（镇）建设。加快林业生态市建设。大力推进四大森林公园建设、“两岭”示范开发和千村绿化工程，完成新造林面积25万亩，努力构筑国家森林城市框架。深入推进新农村建设。全力抓好林州城乡一体化试验示范县、18个省级示范中心村和14个市级“六位一体”整体推进示范区规划建设。完成90个贫困村整村推进和4.3万贫困人口脱贫任务。继续抓好农村沼气和乡村清洁工程，新增农村沼气用户3万户，解决15万农民用气问题。加大农村饮水安全工程建设，解决25万人饮水安全问题。

    （四）扎实推进城乡基础设施建设。继续坚持中心城市带动，突出产业为基、就业为本，积极推进现代城镇体系建设。深入推进“城建三年行动计划”。重点实施京港澳高速南北出入口、人民大道区域改造工程。开工建设文峰大道东西段、中华路南北段，构建贯通南北、连接东西的城市交通新干线。完成“六桥一互通”工程，启动石武高铁安阳东站及站前广场建设。加快城市水系规划建设，大力实施洹河水系景观、洪河开发区段、护城河综合治理和东区中轴线水系建设工程，启动南水北调调蓄池及管线配套工程，着力改善人居环境。大力实施“现代交通体系行动计划”。完成投资58亿元，重点建设京港澳高速扩建、林长高速、鹤辉高速、石武高铁、山西中南部铁路通道安阳段和西南外环线等工程。积极谋划和适时推进西北环城高速、石林高速、滑县快速通道前期工程。加快航空港、陆路港、“八挂来网”、客货运集团、现代物流等项目建设，构建现代综合交通运输体系，推动安阳枢纽型城市全面协调发展。切实加强城市基础设施建设与管理。开展控制性详细规划等规划编制，进一步完善在新版城市总体规划指导下的规划编制体系。基本完成全市县域村镇体系规划编制，推进城乡规划全覆盖，努力形成中心城市、中等城市、小城镇和农村社区层次分明、结构合理的现代城镇体系。统筹布局和整合全市产业、城镇、交通、生态等各类空间要素，合理安排县域城镇建设、农田保护、产业集聚、村落分布、生态涵养等空间布局。积极开展城市重要主干道街景整治和亮化美化工作。完善安东新区路网配套设施建设，积极推进城市公共交通、供水、供热、燃气、污水、垃圾处理等基础设施建设。加快数字化城管体系建设，改革城管体制，提高城市基础设施运行效率。加快推进城乡一体化建设。强化户籍管理和社会保障制度改革，放宽县城、小城镇落户条件，进一步提高城镇化率。加快小城镇基础设施和公共设施建设，确保全市小城镇镇区常住人口增长2.5%以上。加大财政转移支付制度，把公共资源更多地投向农村，促进城市基础设施和公共设施向农村延伸，构建城乡一体、资源共享的公共服务体系，把我市建成在中原城市群中地位领先、在周边地市中具有明显竞争力的豫北区域性中心强市。

    （五）扎实推进文化旅游业。充分发挥文化旅游业在扩内需、保增长、调结构中的独特作用，围绕打造文化旅游强市，积极实施“文化旅游行动计划”。加快文化产业发展。实施文化精品工程，打造文化品牌，提升文化产业实力和竞争力。办好2010中国？安阳殷商文化旅游节、“欢乐中原”广场文艺演出等活动。积极申办中国文字节。加快殷墟国家大遗址公园建设，完成殷墟保护总体规划修编和报批工作。切实做好曹操高陵的保护、发掘与利用工作。启动中国文字博物馆二期工程和羑里中华周易文化苑项目建设。加快动漫等新兴文化产业发展。支持深圳华强集团建设安阳影视基地，拍摄反映殷商文化的影视作品。加快旅游业发展。以“探奇？安阳”为核心价值，深入实施“三阳开泰”旅游发展战略，打造殷墟、红旗渠、中国文字博物馆、羑里城、二帝陵、曹操高陵、马氏庄园等品牌。推进殷墟、红旗渠与林虑山5A景区创建工作。积极打造“中国航空运动之都”。筹建国家级自驾车营地。深化文化体制改革。全面完成经营性文化事业单位和国有文艺院团转企改制任务，积极组建报业和演艺两大集团，鼓励建设文化投资公司，扎实推进县（市、区）文化管理体制改革。切实加强精神文明建设。深入推进社会主义核心价值体系建设，大力弘扬以爱国主义为核心的民族精神和以改革创新为核心的时代精神，全面提升市民素质。积极创造条件争取创建全国文明城市。加强诚信体系建设，深入开展“三优三创”竞赛活动和以“清洁家园行动”为载体的农村精神文明创建活动。加强未成年人思想道德建设，进一步净化社会文化环境。

    （六）扎实推进改革开放。着眼于解决制约科学发展、跨越发展的突出矛盾和问题，把改革创新和对外开放向纵深推进，进一步增强经济社会发展的活力和动力。深入推进各项改革。实施“重点改革行动计划”，着力构建充满活力、富有效率、有利于科学发展的体制机制。加快国企改革步伐，努力完成国企改革攻坚任务。坚持有进有退原则，做大做强市属国有经济。完善国有资产监管体制，确保国有资产保值增值。积极推进农村改革，基本完成集体林权制度改革，开展农村土地承包经营权流转改革。实施医药卫生体制改革，完善基本医疗保障制度，全面实现医疗救助与新农合、城镇职工和居民医保同步结算。城镇居民医保和新农合财政补助标准提高到每人每年120元。继续推进文化体制改革。稳步推进资源性价格和环保收费改革。积极做好财税、投资、教育、行政管理等领域的改革。大力发展非公有制经济。继续鼓励、支持、引导非公有制经济快速发展，全面清理融资、土地使用等公共服务方面的歧视性法规政策，促进公平竞争。全力提高开放型经济水平。积极实施“对外开放行动计划”，深入开展大招商活动，不断拓展对外开放的广度和深度。突出县（市、区）和企业招商主体，瞄准国内外500强、行业500强企业，围绕九大产业集聚区和七大主导产业开展招商引资。积极参加全国和全省重要经贸活动。筹备好珠三角光伏产业、侨商安阳行、韩资企业等专题招商活动。做好境外招商活动。积极开拓国际市场，落实和完善各项出口政策，培育一批特色明显、具有较强国际竞争力的骨干企业和拳头产品。不断优化开放环境，推行外来项目无偿代理制，建立健全服务平台。

    （七）扎实推进民生民心工程。坚持把民生工作摆在更加突出的位置，集中财力办好以十个方面实事为基础的“十项民生工程”，下大力气解决好事关人民群众切身利益的现实问题。千方百计扩大就业。大力实施“就业创业行动计划”，继续实行积极的就业政策，延续执行“三缓、三减、三补贴”优惠政策。强化对高校毕业生、下岗失业人员、“4050”人员和农民工的就业服务，大力开发各类就业岗位特别是公益性就业岗位。动态消除零就业家庭。实现失业人员再就业3万人，就业困难群体再就业1.1万人，应届高校毕业生就业率达到80%以上，新增转移就业农村劳动力6万人。加大小额担保贷款工作力度，发放小额担保贷款2亿元。不断提高社会保障和住房保障水平。继续扩大企业养老、失业、工伤和生育保险覆盖面，着力解决关闭破产企业退休人员和困难企业职工的医疗保障问题。做好内黄县全国新农保试点工作，适时在全市范围内推广。继续提高企业退休人员养老金。完善落实城乡居民最低生活保障制度，加强对困难群众的社会救助。加大保障性住房建设，为城市低收入家庭提供2000套经济适用房。开工建设廉租住房33.4万平方米，竣工18.1万平方米，为全市低收入住房困难家庭提供3620套廉租住房。加快发展各项社会事业。以实施全面素质教育为重点，大力推进义务教育均衡发展。积极发展职业教育，完善高等教育结构。继续对全市义务教育阶段学生免除学杂费，免费为农村中小学生提供教科书，并为农村贫困家庭寄宿生提供生活费补助。继续实施农村初中校舍改造、中小学校舍安全工程，启动城镇中小学校新建、改扩建工程。完成中医药学校整体搬迁。加快市职业教育中心、东区小学等项目建设。积极推进市实验中学新建教学楼、市第八中学搬迁等项目建设。加快卫生事业发展，推进社区卫生服务中心规范化建设。力争2—3年内完成所有村卫生室建设改造任务。继续做好甲型H1N1流感等重大传染病防控工作，稳步推进新农合门诊统筹工作。全力创建国家卫生城市。加强体育基础设施建设，研究制定《全民健身实施计划》，认真办好世界杯滑翔伞比赛，做好省十一届运动会参赛工作。进一步完善人才政策，吸引和用好各类人才。提高出生人口素质，有效遏制出生人口性别比例升高势头，争创全国人口和计划生育综合改革示范市。依法搞好第六次全国人口普查。全力维护社会稳定。积极实施“安全发展行动计划”，继续开展“平安安阳”创建活动。深入开展重点领域隐患治理，坚决遏制重特大事故发生。加强社会治安综合治理，依法防范和打击各类违法犯罪活动，确保社会大局和谐稳定。完善应急管理机制，积极预防和妥善处置群体性事件和突发公共安全事件。积极开展法律服务和法律援助。加强社区建设，完善村民自治。

    同时，继续做好国防教育和民兵预备役建设，深入开展双拥共建活动，巩固发展军政军民团结。加大对地下空间的开发利用，完善人防工程，搭建政府应急平台。充分发挥工会、共青团、妇联等人民团体的桥梁纽带作用。切实做好民政、统计、审计、地震、史志、经济研究、外事侨务、民族宗教、档案管理等工作。完成援川灾后重建任务。力争全面完成“十一五”规划各项目标任务。编制好“十二五”规划。

    （八）扎实推进政府自身建设。深入开展学习实践科学发展观活动，扎实推进“两转两提”，立足于学、立足于做、立足于实、立足于效，努力打造学习型、服务型、法治型、廉洁型政府。始终坚持依法行政。严格执行人大及其常委会的决议、决定，依法接受人大及其常委会的法律监督、工作监督，主动接受政协民主监督，认真办理人大代表建议和政协委员提案。强化行政复议，规范执法行为。完善行政问责制度，坚决纠正行政不作为和乱作为。切实转变政府职能。深化行政管理体制改革，进一步减少和规范行政审批。创新行政服务中心管理机制，做到一个机构对外、一个机构履职、一枚印章审批。加强电子政务建设，启动网上行政审批一期工程，建成覆盖市县两级的行政效能电子监察系统。继续开展政风行风评议，对市县政府部门内设科、股负责人进行监督考评。创新轮岗交流和竞争上岗机制。大力整治文风会风，严格控制各类检查评比和节庆论坛活动。加强学习型机关建设，深入开展争做“人民满意的公务员”活动，不断提高公务员队伍整体素质。进一步强化服务意识。尊重群众的主体地位，作决策、编规划、定政策站在群众的角度换位思考，问政于民、问需于民，充分调动人民群众参与改革发展的积极性、主动性和创造性。继续办好市长热线和效能监督热线，想群众之所想、急群众之所急、办群众之所需、解群众之所难，多做雪中送炭、排忧解愁的实事。着力改进工作作风。坚持求真务实，坚决克服形式主义、官僚主义，反对急功近利、虚报浮夸。尊重规律，科学决策，努力做到不走错路、少走弯路。完善考核评价机制，牢固树立正确的政绩观，说到做到、说好做好，实干实效。艰苦奋斗，勤俭节约，严格控制一般性支出，从严控制公费出国出境，坚决制止铺张浪费和奢靡之风。更加注重廉政建设。坚持标本兼治、综合治理、惩防并举、注重预防的方针，落实廉政建设责任制。健全公共资源配置、公共资产交易、公共产品生产等领域的管理制度，推进权利运行程序化，从源头上防治腐败。依法推进政务公开，主动接受各方面监督，让权力在阳光下运行。逐步将公务消费纳入政务公开范围，探索建立系统规范的财政预算公开机制。

各位代表！

    时代赋予重任，奋斗铸就辉煌。任何困难都改变不了我们跨越式发展的强烈愿望，任何挑战都阻挡不住我们跨越式发展的坚定步伐。让我们紧密团结在以胡锦涛同志为总书记的党中央周围，在省委、省政府和市委的坚强领导下，全面贯彻落实科学发展观，坚定信心，昂扬奋进，万众一心，攻坚克难，奋力夺取经济社会又好又快发展新的胜利！
